# Novel Computational Tools in Biosignal Processing

**DOI:** 10.1155/2015/984702

**Published:** 2015-04-19

**Authors:** Gaoxiang Ouyang, Tania Stathaki, Hak-Keung Lam, Jing Li, Zhaojie Ju

**Affiliations:** ^1^National Key Laboratory of Cognitive Neuroscience and Learning, Beijing Normal University, Beijing 100875, China; ^2^Department of Electrical and Electronic Engineering, Imperial College London, London SW7 2AZ, UK; ^3^Department of Informatics, King's College London, London WC2R 2LS, UK; ^4^School of Information Engineering, Nanchang University, Nanchang 330031, China; ^5^Intelligent Systems and Biomedical Robotics Group, University of Portsmouth, Portsmouth PO1 3HE, UK

Biosignal processing has become a popular part of modern computational science, physics, and biological science. It is desired to develop novel computational tools to analyze biosignals, which are able to provide utilizable approaches to enhance our understanding of complex biological systems.

Novel methods were proposed for analyzing biosignals, such as electroencephalogram (EEG) and electrocardiogram (ECG), which might provide researchers with the current state-of-the-art knowledge of this interdisciplinary research field.

The paper titled “Dual Adaptive Filtering by Optimal Projection Applied to Filter Muscle Artifacts on EEG and Comparative Study” by S. Boudet et al. proposes a dual adaptive filtering by optimal projection (DAFOP) method to automatically remove artifacts on EEG recordings. It is demonstrated that the DAFOP is significantly more efficient than the canonical correlation analysis (CCA) and conventional low-pass filtering methods.

The paper titled “Study of Driving Fatigue Alleviation by Transcutaneous Acupoints Electrical Stimulations” by F. Wang and H. Wang investigates the driving fatigue by calculating the relative power spectrum of drivers' EEG signals, which were collected from the drivers who were electrically stimulated and the drivers without electrical stimulation. They found that the electrical stimulation method stimulating the Láogóng point of the human body can alleviate driving fatigue effectively.

The paper titled “Cortical Source Multivariate EEG Synchronization Analysis on Amnestic Mild Cognitive Impairment in Type 2 Diabetes” by D. Cui et al. investigates the synchronization in five ROIs of sLORETA sources for seven bands in amnestic mild cognitive impairment (aMCI) and normal cognitive functions subjects in type 2 diabetes mellitus (T2DM). It is found that the cortical source synchronization is significantly different between aMCI and control group, and this difference is correlated with cognitive functions.

The paper titled “Detection of Burst Suppression Patterns in EEG Using Recurrence Rate” by Z. Liang et al. uses recurrent plot (RP) analysis for the detection of the burst suppression pattern (BSP) in EEG. Their results suggest that the proposed RP may provide an effective burst suppression detector for developing new patient monitoring systems.

The paper titled “Enhanced Performance by Time-Frequency-Phase Feature for EEG-Based BCI Systems” by B. Xu et al. introduces a new motor parameter imagery paradigm using clench speed and clench force motor imagery. It is demonstrated that the proposed method has the potential to increase the direct control commands for BCI control and the time-frequency-phase feature has the ability to improve BCI classification accuracy.

The paper titled “Sparse Matrix for ECG Identification with Two-Lead Features” by K.-K. Tseng et al. proposes a new two-dimensional sparse matrix to identify human ECG with higher accuracy.

The paper titled “Multiple-Site Hemodynamic Analysis of Doppler Ultrasound with an Adaptive Color Relation Classifier for Arteriovenous Access Occlusion Evaluation” by J.-X. Xu proposes an adaptive color relation classifier to screen the degrees of stenosis for an arteriovenous access occlusion evaluation by multiple-site hemodynamic analysis of Doppler ultrasound. Their results show that the proposed screening method efficiently evaluates access occlusion.

The paper titled “An Effective Measured Data Preprocessing Method in Electrical Impedance Tomography” by C. Yu et al. proposes a nonlinear transformation to improve the electrical impedance tomography (EIT) spatial resolution.

As we have seen, biosignal processing is a very active area, and many challenges still remain. It can be expected that more exciting research activities will be seen in the near future. Hopefully, this special issue will contribute in diffusing novel computational tools of biosignal processing to various application fields.

